# Topographical Response of Retinal Neovascularization to Aflibercept or Panretinal Photocoagulation in Proliferative Diabetic Retinopathy: Post Hoc Analysis of the CLARITY Randomized Clinical Trial

**DOI:** 10.1001/jamaophthalmol.2021.0108

**Published:** 2021-03-11

**Authors:** Sandra Halim, Manjula Nugawela, Usha Chakravarthy, Tunde Peto, Savita Madhusudhan, Pauline Lenfestey, Barbara Hamill, Yalin Zheng, David Parry, Luke Nicholson, John Greenwood, Sobha Sivaprasad

**Affiliations:** 1 National Institute for Health Research Biomedical Research Centre, Moorfields Eye Hospital National Health Service Foundation Trust, London, United Kingdom; 2 Institute of Ophthalmology, University College London, London, United Kingdom; 3 Institute of Clinical Science, Center for Experimental Medicine, Queen’s University, Belfast, United Kingdom; 4 St Paul’s Eye Unit, Royal Liverpool University Hospital, Liverpool, United Kingdom

## Abstract

**Importance:**

Eyes with proliferative diabetic retinopathy have a variable response to treatment with panretinal photocoagulation (PRP) or anti–vascular endothelial growth factor agents. The location of neovascularization (NV) is associated with outcomes (eg, patients with disc NV [NVD] have poorer visual prognosis than those with NV elsewhere [NVE]).

**Objective:**

To investigate the distribution of NV in patients with proliferative diabetic retinopathy and the topographical response of NV to treatment with aflibercept or PRP.

**Design, Setting, and Participants:**

This post hoc analysis of the phase 2b randomized clinical single-masked multicenter noninferiority Clinical Efficacy and Mechanistic Evaluation of Aflibercept for Proliferative Diabetic Retinopathy (CLARITY) trial was conducted from November 1, 2019, to September 1, 2020, among 120 treatment-naive patients with proliferative diabetic retinopathy to evaluate the topography of NVD and NVE in 4 quadrants of the retina on color fundus photography at baseline and at 12 and 52 weeks after treatment.

**Exposures:**

In the CLARITY trial, patients were randomized to receive intravitreal aflibercept (2 mg/0.05 mL at baseline, 4 weeks, and 8 weeks, and as needed from 12 weeks onward) or PRP (completed in initial fractionated sessions and then on an as-needed basis when reviewed every 8 weeks).

**Main Outcomes and Measures:**

Main outcomes were per-retinal quadrant frequencies of NV at baseline and frequencies of patterns of regression, recurrence, and new occurrence at 12-week and 52-week unmasked follow-up.

**Results:**

The study included 120 treatment-naive patients (75 men; mean [SD] age, 54.8 [14.6] years) with proliferative diabetic retinopathy (there was a 1:1 ratio of eyes to patients). At baseline, NVD with or without NVE was observed in 42 eyes (35.0%), and NVE only was found in 78 eyes (65.0%); NVE had a predilection for the nasal quadrant (64 [53.3%]). Rates of regression with treatment were higher among eyes with NVE (89 of 102 [87.3%]) compared with eyes with NVD (23 of 43 [53.5%]) by 52 weeks, with NVD being more resistant to either treatment with higher rates of persistence than NVE (20 of 39 [51.3%] vs 29 of 100 [29.0%]). Considering NVE, the regression rate in the temporal quadrant was lowest (32 of 42 [76.2%]). Eyes treated with aflibercept showed higher rates of regression of NVE compared with those treated with PRP (50 of 52 [96.2%] vs 39 of 50 [78.0%]; difference, 18.2% [95% CI, 5.5%-30.8%]; *P* = .01), but no difference was found for NVD (11 of 17 [64.7%] vs 12 of 26 [46.2%]; difference, 18.6% [95% CI, −11.2% to 48.3%]; *P* = .23).

**Conclusions and Relevance:**

This post hoc analysis found that NVD is less frequent but is associated with more resistance to currently available treatments than NVE. Aflibercept was superior to PRP for treating NVE, but neither treatment was particularly effective against NVD by 52 weeks. Future treatments are needed to better target NVD, which has poorer visual prognosis.

**Trial Registration:**

isrctn.org Identifier: ISRCTN32207582

## Key Points

**Question:**

What is the topographic distribution of retinal neovascularization in patients with proliferative diabetic retinopathy, and is there an association between response to treatment and the retinal location of the neovascularization?

**Findings:**

In this post hoc unmasked analysis of the Clinical Efficacy and Mechanistic Evaluation of Aflibercept for Proliferative Diabetic Retinopathy (CLARITY) randomized clinical trial, treatment-naive retinal neovascularization elsewhere (NVE) had a predilection for the nasal quadrant. By 52 weeks, the aflibercept group was more likely to have regression of NVE; neither treatment was likely to be associated with complete regression of disc neovascularization (NVD).

**Meaning:**

This study further elaborates on the outcomes of the CLARITY trial in that, anatomically, despite the superior outcomes among patients within NVE who received aflibercept, regression was seen more in NVE than in NVD.

## Teaser

This post hoc analysis of the CLARITY randomized clinical trial investigates the distribution of neovascularization in patients with proliferative diabetic retinopathy and the topographical response of neovascularization to treatment with aflibercept or panretinal photocoagulation.

## Introduction

Proliferative diabetic retinopathy (PDR) is a common cause of visual impairment in people with diabetes. It is characterized by neovascularization (NV) of the optic nerve head and/or anywhere in the retina. Based on 7 fields and color stereoscopic fundus photographs, high-risk PDR is defined as moderate to severe NV (>1/4 to 1/3 disc diameter) of the disc (NVD), moderate NVD (<1/4 to 1/3 disc diameter), or moderate NV elsewhere (NVE) with vitreous or preretinal hemorrhage.^1^ Panretinal photocoagulation (PRP) has been the criterion standard treatment for high-risk PDR for more than 40 years^2^ and, on an individual basis, is also used to treat low-risk PDR and very severe nonproliferative diabetic retinopathy. Although the exact mechanism of PRP is unclear, destruction of areas of retinal capillary nonperfusion (CNP) is hypothesized to reduce the angiogenic drive from these areas. Repeated sessions of PRP may be required to cause regression of these new vessels, suggesting that some new vessels are more resistant to regression than others.^2^

In a trial conducted by the Diabetic Retinopathy Clinical Research network designed to evaluate the comparative efficacy of anti–vascular endothelial growth factor (VEGF) agents vs PRP for PDR, improvements in Diabetic Retinopathy Severity Scale severity and a lower risk of vitrectomy and new-onset diabetic macular edema were reported at 5 years for participants with PDR who received anti-VEGF agents compared with those who received PRP.^3,4^ The Clinical Efficacy and Mechanistic Evaluation of Aflibercept for Proliferative Diabetic Retinopathy (CLARITY) trial,^2^ which was specifically designed to evaluate the efficacy of aflibercept (an anti-VEGF therapy) vs PRP for persons with PDR without diabetic macular edema, showed that NV regresses rapidly with aflibercept and that subsequent withholding of treatment resulted in recurrence of NV in a proportion of eyes. Similar to PRP, the patterns of regression of NV can vary. Approximately one-fourth of eyes in the CLARITY trial continued to exhibit active NV during anti-VEGF treatment. Moreover, even in eyes in which NV was seen to have regressed, both reperfusion of the previously closed vessels or proliferation of NV de novo at another location in the retina were observed when treatment was withheld.^2^ Although the retinal vascular geometry and the distribution of CNP have been examined as biomarkers for the development of NV,^5,6,7^ to our knowledge, there has been no systematic study of regression and recurrence patterns by location of NV and whether there is a differential response to treatment based on the location of the NV.

In this post hoc analysis, we describe the regression patterns of NV by retinal location after treatment with either aflibercept or PRP in a subset of treatment-naive eyes with PDR at enrollment to better classify NV, understand treatment targets, and ultimately improve visual prognosis.

## Methods

This is a post hoc analysis of the CLARITY trial (ISRCTN32207582), which was approved by the National Research Ethics Committee London–South East. The post hoc analysis, conducted from November 1, 2019, to September 1, 2020, was designed at the initial stages of enrollment and was approved by the original ethics approval. It is a post hoc analysis because, although it was planned, it was specified after data were available.

The CLARITY trial is a phase 2b randomized clinical, single-masked, multicenter noninferiority trial that compared the 1-year visual function outcomes of aflibercept vs PRP for the treatment of PDR in patients with type 1 or 2 diabetes. Patients aged 18 years or older with treatment-naive or laser-treated active PDR without diabetic macular edema were recruited from 22 UK ophthalmic centers and randomly assigned to intravitreal aflibercept (2 mg/0.05 mL at baseline, 4 weeks, and 8 weeks, and as needed from 12 weeks onward) or PRP (completed in initial fractionated sessions and then on an as-needed basis when reviewed every 8 weeks). Retreatment criteria were based on regression pattern with additional doses required if evidence of active NV, which was defined as recurrence, persistence, or new occurrence. Patients were followed up for 52 weeks. The CLARITY trial has been published separately.^2^

The presence of NVD and NVE was detected on color fundus photography (7-field with either Topcon TRC 50DX [Topcon Healthcare] or Zeiss FF450 Plus [Zeiss Medical Technology], or ultra-widefield [UWF] using the Optos 200Tx [Optos]) of the study eye captured to a standardized protocol at baseline, 12 weeks, and 52 weeks (eTable 1 in the Supplement). Only eyes that were treatment naive at baseline were included in the present analysis. Participants randomized to the aflibercept group received mandated injections (2 mg/0.05 mL per injection) at baseline, 4 weeks, and 8 weeks. From 12 weeks onward, injections were given as needed every 4 weeks based on disease progression, and supplemental PRP was performed if there was no regression of NV. Injections were deferred if eyes had adverse events, such as vitreous hemorrhage, retinal detachment, or increased intraocular pressure of greater than 30 mm Hg. Participants were switched to PRP if aflibercept became contraindicated during the trial period (eg, pregnancy or blood clot). Those randomized to the PRP group received direct or indirect or single or multispot laser treatment, targeting areas of nonperfusion at baseline and in fractionated sessions every 2 weeks until week 12. From week 12, they were followed up every 8 weeks. Panretinal photocoagulation was deferred at investigator discretion if the media were too hazy or if the eye had received adequate PRP.

### Grading of Color and Fluorescein Angiographic Images

Anonymized images captured at baseline, 12 weeks, and 52 weeks with no accompanying clinical information were uploaded to a secure server and graded to a prespecified protocol by trained staff at the Network of Reading Centers in the United Kingdom (NetwORC; Belfast, Moorfields, and Liverpool). A bespoke grid (NetWORC UK grid) centered on the fovea of UWF or 7-field montage images was used, dividing the posterior pole and beyond into 4 quadrants (superior, temporal, inferior, and nasal) to describe the topography of the new vessels. We excluded all eyes with previous PRP or with inadequate image quality due to cataract, vitreous hemorrhage, eyelashes, eyelid, or artifacts interfering with the grading of the UWF images. When the far peripheral retina was not clearly visible, only the gradable retina was used for analysis.

### Regression and Recurrence Patterns by Location of NVD and NVE on Color Imaging

For NVD, presence and absence were graded. For NVE, both presence and location were documented by quadrant. Treatment response within the quadrants was categorized into the following 4 groups: (1) regressed NV, defined as the complete absence of NV or a reduction in the area of fundus affected by NV as per the grader’s assessment from the previous visit on color fundus photographs (fibrotic NV was counted as absence of NV); (2) de novo occurrence of NV within a quadrant or disc since the previous visit when previously there was none; (3) persistent NV, where there is no change or increase in the area of NV from the previous visit; and (4) for week 52 analysis, we defined a further category of recurrence after initial regression observed at week 12 (eTable 2 in the Supplement).

### Statistical Analysis

The presence of NVD and the location of NVE by quadrant at baseline in treatment-naive eyes with PDR in the 2 groups were compared using Pearson χ^2^ tests. Regression rates of NVD and NVE by quadrant at 12 and 52 weeks were described as percentage frequencies, and the frequencies between the 2 treatment groups were compared using the Pearson χ^2^ test. The frequency denominators in Table 1 and Table 2 signify the number of eyes eligible for the particular outcome, so, for regression, the number of eyes eligible would be the number of eyes with NV present at baseline or week 12; for persistence, the number of eyes eligible would be the number of eyes with NV present at baseline or week 12; for new occurrence, the number of eyes eligible would be the number of eyes without NV at baseline or week 12; and for recurrence, the number of eyes eligible would be the number of eyes that regressed at week 12. Treatment response between the 2 treatment groups for each location was compared using descriptive risk differences with 95% CIs. Treatment response of NVD and NVE was compared using the Pearson χ^2^ test and descriptive risk difference with 95% CIs. All statistical analyses were performed using Stata software, version 15.1 (StataCorp). All *P* values are 2-sided, and results were deemed statistically significant at *P* < .05; no adjustments were made for multiple analyses.

**Table 1.** Treatment Outcome at Week 12 per Treatment Group

Outcome at week 12	Eyes, No./total No. (%)^a^		Difference, % (95% CI)	*P* value
	PRP (n = 61)	Aflibercept (n = 59)		
Regression				
Superior	14/23 (60.9)	19/23 (82.6)	21.7 (−3.5 to 47.0)	.10
Inferior	7/13 (53.8)	9/12 (75.0)	21.2 (−15.4 to 57.7)	.27
Nasal	14/27 (51.9)	31/38 (81.6)	29.7 (7.2 to 52.2)	.01
Temporal	12/22 (54.5)	12/20 (60.0)	5.5 (−24.4 to 35.4)	.72
Any NVE	35/50 (70.0)	48/52 (92.3)	22.3 (7.7 to 36.9)	.004
Disc	7/26 (26.9)	9/17 (52.9)	26.0 (−3.2 to 55.2)	.08
Any site	41/58 (70.7)	52/58 (89.7)	19.0 (4.9 to 33.1)	.01
New occurrence				
Superior	3/41 (7.3)	1/37 (2.7)	−4.6 (−14.1 to 4.9)	.36
Inferior	1/49 (2.0)	1/48 (2.1)	<0.1 (−5.6 to 5.7)	.99
Nasal	1/35 (2.9)	0/21 (0)	−2.9 (−8.4 to 2.7)	.43
Temporal	6/45 (13.3)	2/41 (4.9)	−8.5 (−20.4 to 3.5)	.18
Any NVE	10/61 (16.4)	4/58 (6.9)	−9.5 (−20.8 to 1.9)	.11
Disc	0/35 (0)	1/43 (2.3)	2.3 (−2.2 to 6.8)	.36
Any site	10/61 (16.4)	5/59 (8.5)	−7.9 (−19.6 to 3.8)	.19
Persistence				
Superior	4/18 (22.2)	2/21 (9.5)	−12.7 (−35.6 to 10.2)	.27
Inferior	3/10 (30.0)	1/10 (10.0)	−20.0 (−53.9 to 13.9)	.26
Nasal	9/23 (39.1)	4/35 (11.4)	−27.7 (−50.3 to −5.1)	.01
Temporal	3/15 (20.0)	5/17 (29.4)	9.4 (−20.2 to 39.1)	.54
Any NVE	17/48 (35.4)	11/52 (21.2)	−14.3 (−31.8 to 3.2)	.11
Disc	13/20 (65.0)	6/15 (40.0)	−25.0 (−57.4 to 7.4)	.14
Any site	26/56 (46.4)	17/58 (29.3)	−17.1 (−34.7 to 0.4)	.06

Abbreviations: NVE, neovascularization elsewhere; PRP, panretinal photocoagulation.

^a^ The denominators represent the number of eyes eligible for the particular outcome, so, for regression, this would be those with neovascularization present at baseline or week 12; for persistence, this would be those with neovascularization present at baseline or week 12; for new occurrence, this would be those without neovascularization at baseline or week 12; and, for recurrence, this would be the number of eyes that regressed at week 12. Because the denominators are different for each group, percentages are not directly comparable between groups and may not add up to 100%. The Pearson χ^2^ test was used to compare the aflibercept group with the PRP group.

**Table 2.** Treatment Outcome at Week 52 for the Whole Cohort and per Treatment Group

Outcome at week 52	Eyes, No./total No. (%)^a^		Difference, % (95% CI)	*P* value
	PRP (n = 61)	Aflibercept (n = 59)		
Regression				
Superior	16/23 (69.6)	22/23 (95.7)	26.1 (5.5 to 46.7)	.02
Inferior	8/13 (61.5)	12/12 (100.0)	38.5 (12.0 to 64.9)	.02
Nasal	16/27 (59.3)	36/38 (94.7)	35.5 (15.6 to 55.3)	<.001
Temporal	15/22 (68.2)	17/20 (85.0)	16.8 (−8.2 to 41.8)	.20
Any NVE	39/50 (78.0)	50/52 (96.2)	18.2 (5.5 to 30.8)	.01
Disc	12/26 (46.2)	11/17 (64.7)	18.6 (−11.2 to 48.3)	.23
Any site	49/58 (84.5)	54/58 (93.1)	8.6 (−2.8 to 20.0)	.14
New occurrence				
Superior	8/41 (19.5)	5/37 (13.5)	−6.0 (−22.4 to 10.4)	.48
Inferior	10/49 (20.4)	6/48 (12.5)	−7.9 (−22.6 to 6.8)	.29
Nasal	7/35 (20.0)	2/21 (9.5)	−10.5 (−28.7 to 7.8)	.30
Temporal	11/45 (24.4)	3/41 (7.3)	−17.1 (−32.0 to −2.3)	.03
Any NVE	24/61 (39.3)	11/58 (19.0)	−20.4 (−36.3 to −4.5)	.01
Disc	3/35 (8.6)	1/43 (2.3)	−6.2 (−16.6 to 4.1)	.21
Any site	25/61 (41.0)	11/59 (18.6)	−22.3 (−38.2 to −6.5)	.01
Persistence				
Superior	5/19 (26.3)	2/22 (9.1)	−17.2 (−40.4 to 5.9)	.14
Inferior	4/11 (36.4)	1/11 (9.1)	−27.3 (−60.4 to 5.8)	.13
Nasal	10/24 (41.7)	4/37 (10.8)	−30.9 (−53.0 to −8.7)	.005
Temporal	3/15 (20.0)	5/18 (27.8)	7.8 (−21.2 to 36.7)	.60
Any NVE	18/48 (37.5)	11/52 (21.2)	−16.3 (−34.0 to 1.3)	.07
Disc	14/23 (60.9)	6/16 (37.5)	−23.4 (−54.4 to 7.6)	.15
Any site	28/58 (48.3)	17/58 (29.3)	−19.0 (−36.4 to −1.6)	.04
Recurrence				
Superior	6/14 (42.9)	2/19 (10.5)	−32.3 (−61.7 to −3.0)	.03
Inferior	3/7 (42.9)	5/9 (55.6)	12.7 (−36.2 to 61.7)	.61
Nasal	7/14 (50.0)	11/31 (35.5)	−14.5 (−45.7 to 16.6)	.36
Temporal	6/12 (50.0)	4/12 (33.3)	−16.7 (−55.5 to 22.2)	.41
Any NVE	18/35 (51.4)	15/48 (31.3)	−20.2 (−41.3 to 0.9)	.06
Disc	2/7 (28.6)	1/9 (11.1)	−17.5 (−56.7 to 21.8)	.38
Any site	0/1 (0)	1/5 (20.0)	20.0 (−15.1 to 55.1)	.62

Abbreviations: NVE, neovascularization elsewhere; PRP, panretinal photocoagulation.

^a^ The denominators represent the number of eyes eligible for the particular outcome, so, for regression, this would be those with neovascularization present at baseline or week 12; for persistence, this would be those with neovascularization present at baseline or week 12; for new occurrence, this would be those without neovascularization at baseline or week 12; and, for recurrence, this would be the number of eyes that regressed at week 12. Because the denominators are different for each group, percentages are not directly comparable between groups and may not add up to 100%. The Pearson χ^2^ test was used to compare the aflibercept group with the PRP group.

## Results

### Study Cohort

Of the 232 patients recruited in the CLARITY trial, 120 treatment-naive patients (51.7%) with PDR who received either aflibercept or PRP were included in this post hoc analysis (Figure). The mean (SD) age of the 120 participants was 54.8 (14.6) years, and 75 patients (62.5%) were men. Sixty-nine patients (57.5%) underwent imaging using Optos UWF imaging, and 51 patients (42.5%) underwent imaging using non-Optos 7-field imaging. The mean (SD) duration of diabetes was 25.2 (10.5) years, with a mean (SD) hemoglobin A_1c_ level of 73.0 (21.1) mmol/mol (8.8% [4.1%] of total hemoglobin [to convert to proportion of total hemoglobin, multiply by 0.01]), and a mean blood pressure of 134/79 mm Hg.

**Figure.**
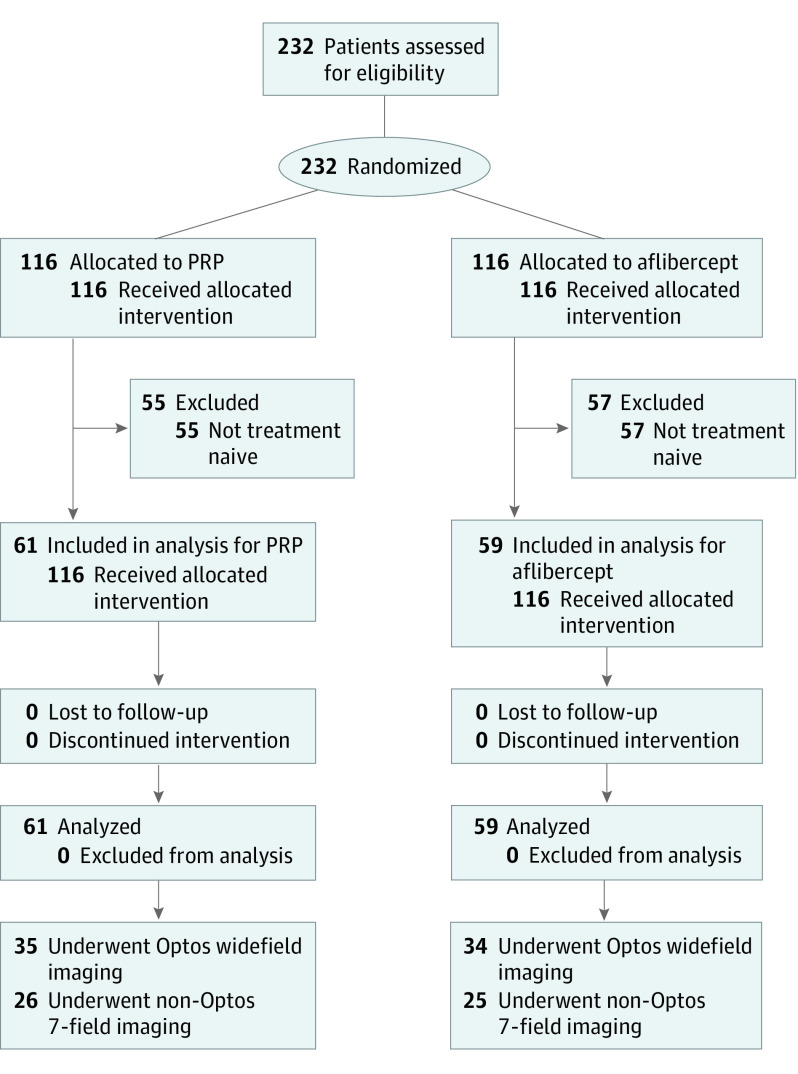
Flowchart of Study Participants PRP indicates panretinal photocoagulation.

### Topography of New Vessels at Baseline

There were more eyes with NVE only (78 [65.0%]) compared with eyes with NVD and NVE (42 [35.0%]). A total of 16 eyes (13.3%) had only NVD. Table 3 shows the proportion of eyes with NVD and NVE by location and by treatment assignment. At baseline, NVE was most frequently seen in the nasal quadrant (64 [53.3%]), followed by the superior (42 [35.0%]), temporal (34 [28.3%]), and inferior (23 [19.2%]) quadrants (Table 3). There was more nasal NVE in the aflibercept group compared with the PRP group (38 of 59 [64.4%] vs 26 of 61 [42.6%]; *P* = .02).

**Table 3.** Frequency of Neovascularization by Treatment Group and by Retinal Location at Baseline

Location	Eyes, No. (%)	
	PRP (n = 61)	Aflibercept (n = 59)
Eyes		
With NVD	26 (42.6)	16 (27.1)
With NVE only	35 (57.4)	43 (72.9)
Eyes with NVE		
Superior	20 (32.8)	22 (37.3)
Inferior	12 (19.7)	11 (18.6)
Nasal	26 (42.6)	38 (64.4)
Temporal	16 (26.2)	18 (30.5)
Eyes with NVE with or without NVD	48 (78.7)	52 (88.1)

Abbreviations: NVD, disc neovascularization; NVE, neovascularization elsewhere; PRP, panretinal photocoagulation.

### Topography of Treatment Response

Table 1 and Table 2 show the regression patterns of NVD and NVE at 12 and 52 weeks, respectively. The proportion of eyes in which NVD regressed was lower, with 16 of 43 (37.2%) graded as regressed at week 12 (Table 1). By contrast, 81.4% of eyes with NVE (83 of 102) were regressed at 12 weeks. At week 52, approximately half the eyes with NVD exhibited regression (23 of 43 [53.5%]) (Table 2). At 52 weeks, the NVE originally present had regressed in 89 of 102 eyes (87.3%), new-onset NVE was seen in 35 of 119 eyes (29.4%), persistence of NVE or NVD was seen in 45 of 116 eyes (38.8%), and persistent NVE was seen in 29 of 100 eyes (29.0%).

On examining the frequency of NVE treatment response by quadrant, regression was least frequent in the temporal sector (24 of 42 eyes [57.1%] regressed at 12 weeks [Table 1] and 32 of 42 eyes [76.2%] at 52 weeks [Table 2]). In the superior quadrant, NVE responded best to treatment because it had the most number of eyes that showed regression with treatment (33 of 46 eyes [71.7%] at 12 weeks [Table 1] and 38 of 46 eyes [82.6%] at 52 weeks [Table 2]) and the least number of eyes in which NV persisted (6 of 39 eyes [15.4%] at 12 weeks [Table 1] and 7 of 41 eyes [17.1%] at 52 weeks [Table 2]) or recurred (8 of 33 eyes [24.2%] at 52 weeks) during follow-up.

### Comparing NVD With NVE Irrespective of Treatment Type

Table 1 and Table 2 also show that there was no difference in regression patterns of NVD between treatment groups at 12 or 52 weeks. The frequency of persistent NVD (20 of 39 [51.3%]) is much higher than that of NVE (29 of 100 [29.0%]) at 52 weeks (Table 4).

**Table 4.** Treatment Outcomes for NVE and NVD at 12 and 52 Weeks

Outcome	Eyes, No./total No. (%) at 12 wk				Eyes, No./total No. (%) at 52 wk			
	NVD	NVE	Difference, % (95% CI)^a^	*P* value	NVD	NVE	Difference, % (95% CI)^a^	*P* value
Regression	16/43 (37.2)	83/102 (81.4)	97.6 (94.4 to 100)	<.001	23/43 (53.5)	89/102 (87.3)	29.0 (−9.6 to 67.6)	.04
New occurrence	1/78 (1.3)	14/119 (11.8)	−15.8 (−24.0 to −7.6)	.67	4/78 (5.1)	35/119 (29.4)	47.8 (−6.3 to 100)	.05
Persistence	19/35 (54.3)	28/100 (28.0)	5.8 (−33.3 to 44.9)	.78	20/39 (51.3)	29/100 (29.0)	−10.3 (−40.0 to 19.4)	.52

Abbreviations: NVD, disc neovascularization; NVE, neovascularization elsewhere.

^a^ The Pearson χ^2^ test was used to compare NVD with NVE.

### Response by Treatment Group

After examination of the response by treatment group, a higher proportion of eyes with NVE showed regression by 12 weeks in the aflibercept group than in the PRP group (48 of 52 [92.3%] vs 35 of 50 [70.0%]; difference, 22.3% [95% CI, 7.7%-36.9%]) (Table 1). At week 52, more aflibercept-treated eyes showed regression of NVE, although not in all sectors (Table 2). With respect to recurrence of NVE after regression, a lower proportion of eyes showed recurrence in the aflibercept group compared with the PRP group (15 of 48 [31.3%] vs 18 of 35 [51.4%]; difference, −20.2% [95% CI, −41.3% to 0.9%]; *P* = .06). This difference in pattern was associated with faster regression in the nasal quadrant with aflibercept than with PRP at 12 weeks (31 of 38 [81.6%] vs 14 of 27 [51.9%]; difference, 29.7% [95% CI, 7.2%-52.2%]; *P* = .01) (Table 1). By 52 weeks, regression in the PRP group was less frequent than in the aflibercept group. However, the temporal quadrant was most resistant to either treatment, and recurrence was also more frequent in the temporal quadrant. With regard to NVD, no differences were seen between treatment groups for regression, persistence, or recurrence at week 12 or at week 52.

## Discussion

Our analysis showed that NVD is less frequent but is associated with more resistance to currently available treatments compared with NVE (Table 4). Although aflibercept was superior to PRP for treating NVE, neither treatment was particularly effective against NVD by 52 weeks. This finding is clinically relevant because it emphasizes the importance of screening for NVD and classifying the severity of PDR, and severity has direct implications on treatment frequency and response. Key observations from our analysis include the finding that, at baseline, there was a higher proportion of eyes with NVE than NVD and that, during follow-up, there were more new occurrences of NVE than NVD.

Furthermore, NVD was associated with more resistance to either PRP or anti-VEGF therapy compared with NVE. Regression of NVD occurred less frequently compared with regression of NVE, which was apparent at both week 12 and week 52. The proportion of eyes with persistent NVD (51.3%) despite treatment was nearly double that of eyes with persistent NVE (29.0%) at week 52. We hypothesize that the stimulus for NVD exceeds the suppression of VEGF by these treatments because NVD did not respond as well despite being more exposed to anti-VEGF compared with NVE owing to the absence of the internal limiting membrane at the optic disc.^8^ Other investigators have reported that the peripapillary deposit of VEGF and a high density of VEGF receptors within the glia and retinal vessels on the anterior optic nerve, rather than just the increase of retinal CNP, may explain the pathogenesis of NVD.^9^ If this is the case, it substantiates our postulation that the amount of VEGF production at the optic disc in eyes with NVD exceeds the suppression of VEGF by these treatment modalities. A difference in the amount of glial tissue within NVDs vs NVEs may also affect the rate of regression with measures to suppress VEGF exposure.^9,10^ Although it is thought that NVD is a surrogate marker of more generalized ischemia, our results may also suggest that these therapeutic options are insufficient when retinal ischemia increases above a certain threshold. Alternatively, NVD development and regression may not be governed solely by VEGF. The origin of NVD may also explain the differential response of NVD to these treatments because some may be of ciliary origin rather than retinal origin.^8^ Although it seems that NVE compared with NVD is initially more reactive to current treatment, especially to aflibercept compared with PRP, with higher rates of regression, there were also higher rates of recurrence of NVE (39.8%) than NVD (18.8%), which was obvious at week 52.

In terms of the topography of NVE at baseline, we found that most eyes had NVE in the nasal quadrant (53.3%), followed by the superior (35.0%), temporal (28.3%), and inferior quadrants (19.2%). Although several reports suggest a temporal predilection of NVE correlating to the temporal watershed zone,^11,12,13^ a study by Jansson et al^9^ is in accord with our observations of an increased frequency of NVE in the nasal retina. A further reason for discrepancies with prior studies is that UWF imaging captures 3 times more retinal surface than the conventional 7-field montage. Although this may explain the discrepancies with previous reports, further studies are required on the topographical location of retinal NV on UWF imaging to confirm our findings. It has previously been reported that the distribution of NV in terms of distances from the optic disc is not random,^12^ with nasal lesions appearing closer to the disc compared with temporal lesions.^9,13^ We did not observe more NVE in the nasal quadrant having any bearing on treatment response. Our data do not provide information on the timing and the natural history of the topography of disease, which will require a prospective study of disease progression.

The topographical response with regression of NVE in all quadrants except the temporal region with intravitreal aflibercept, with fewer eyes in the PRP group exhibiting similar levels of regression, supports the view that anti-VEGF therapy represents an improved form of treatment. This is important because the temporal retina is the watershed zone and therefore is most vulnerable to ischemia.^14^

The exact reason for rapid regression and less likelihood of NVE recurrence with aflibercept compared with PRP can only be hypothesized. It may be that the destruction of the peripheral retina by PRP to reduce VEGF production is indirect and takes time to become established, which is in contrast to the direct suppression of VEGF by aflibercept. Alternatively, VEGF suppression by ablation of the peripheral retina may be insufficient to cause rapid regression of NV and is in accordance with clinical practice in which supplemental PRP is required to achieve the therapeutic threshold for continued VEGF suppression. A further reason could be that aflibercept blocks all VEGF isoforms and the placental growth factor, and this may explain its added benefit compared with PRP. This possibility was substantiated by the results of Protocol T, in which the regression of NV was better achieved with aflibercept than other anti-VEGF agents.^15^ However, although aflibercept does not improve CNP, the main trigger for NV,^6^ it may stabilize progression of CNP.^16^

### Strengths and Limitations

This study has some strengths, including that it uses data from the CLARITY trial, which is a prospective, 52-week randomized clinical trial. The grading protocol was predefined; grading was carried out independently in an accredited reading center without any accompanying information on treatment.This study also has some limitations. The relatively short 52-week duration of our study does not reflect the long-term treatment needs of PDR. The natural history of the disease may therefore affect the longer-term outcomes of these interventions. The sample size for eyes with NVD was also smaller than those with NVE and NVD. Although this difference reflects real-life epidemiologic patterns, a larger NVD sample size might realize a treatment response. We also did not statistically adjust for the higher proportion of nasal NVE in the aflibercept group compared with the PRP group at baseline, which may have affected our findings of faster regression in the nasal quadrant with aflibercept at 12 weeks. Furthermore, the lack of treatment effect for eyes with NVD may have been caused by the relatively smaller number of eyes with NVD than with NVE at baseline; a larger sample size would be ideal to confirm our findings. Ideally, all eyes should undergo imaging using the same modality; however, we offset the use of different imaging modalities by zooming the Optos images to improve the ability to grade NVD.

## Conclusions

Our study further elaborates the results of the CLARITY trial, which showed that aflibercept is superior to PRP in that there are differences in the rate of regression, recurrence, persistence, and new occurrence between NVD and NVE and that neither treatment is particularly effective for NVD in the short term. We also observed variation in the aforementioned outcomes that were associated with both topography and treatment regimen.

## Notes

Supplement. eTable 1. Numbers of Eyes Which Received 7-Field Imaging vs UWF Imaging eTable 2. Definition of Regression Patterns
