# Supplementary material for: Topographical Response of Retinal Neovascularization to Aflibercept or Panretinal Photocoagulation in Proliferative Diabetic Retinopathy: Post Hoc Analysis of the CLARITY Randomized Clinical Trial
Source: JAMA Ophthalmol. 2021 Mar 11;139(5):1–8. doi: 10.1001/jamaophthalmol.2021.0108 (PMC7953330; doi:10.1001/jamaophthalmol.2021.0108)
Supplement: Supplement. — eTable 1. Numbers of Eyes Which Received 7-Field Imaging vs UWF Imaging eTable 2. Definition of Regression Patterns [file jamaophthalmol-e210108-s001.pdf]

Supplementary Online Content

Halim S, Nugawela M, Chakravarthy U, et al. Topographical response of retinal neovascularization to aflibercept or panretinal photocoagulation in proliferative diabetic retinopathy: post hoc analysis of the CLARITY randomized clinical trial. *JAMA Ophthalmol.* Published online March 11, 2021. doi:10.1001/jamaophthalmol.2021.0108

eTable 1. Numbers of Eyes Which Received 7-Field Imaging vs UWF Imaging
eTable 2. Definition of Regression Patterns

This supplementary material has been provided by the authors to give readers additional information about their work.

© 2021 Halim S et al. *JAMA Ophthalmology*.

eTable 1. Numbers of Eyes Which Received 7-Field Imaging vs UWF Imaging

Imaging modality	Treatment		
	PRP	aflibercept	Total
Non-optos 7 field	26	25	51
Optos wide field	35	34	69
Total	61	59	120

© 2021 Halim S et al. *JAMA Ophthalmology*.

eTable 2. Definition of Regression Patterns

Regression pattern	Definitions of regression patterns
Regression	Any one or more of the following:
	A. Complete regression of NVE/D
	B. Persistent active NV but a decrease in size or density of NV from the previous visit
Persistence	No decrease in size or density of active NV
De novo occurrence	De novo active NV (flat or elevated) where previously there was none
Recurrence	Recurrence can occur at any visit from week 12 and is defined as one or more of the following:
	A. Recurrence / reactivation of NV
	B. De novo NV (flat or elevated) following at least 8 weeks of complete regression

© 2021 Halim S et al. *JAMA Ophthalmology*.
